# Sonographic measurement of renal size in patients undergoing chronic hemodialysis: Correlation with residual renal function

**DOI:** 10.3892/etm.2014.1560

**Published:** 2014-02-19

**Authors:** WU-XING ZHANG, ZHI-MIN ZHANG, BING-SHENG CAO, WEI ZHOU

**Affiliations:** 1 Department of Nephrology, PLA Center of Transplantation, PLA 309th Hospital, Beijing 100091, P.R. China; 2 Department of Ultrasound, PLA 309th Hospital, Beijing 100091, P.R. China

**Keywords:** renal size, renal length, ultrasonography, residual renal function, end-stage renal disease

## Abstract

Previous studies have reported that renal size may change when the function is compromised. However, it is not known whether sonographically measured renal size reflects the residual renal function (RRF) in patients undergoing chronic hemodialysis. A total of 140 patients undergoing chronic hemodialysis (≥3 months) were investigated in the present study. The patients were divided into two groups according to the daily urine volume: Individuals with RRF (RRF+ group; ≥200 ml; n=65) and without RRF (RRF− group; <200 ml; n=75). Renal sizes were measured using sonography and renal volumes were calculated with the ellipsoid formula. Univariable and multivariable stepwise forward logistic regression analyses were performed to examine the correlation between the presence of RRF and various variables. The results indicated that there were statistically significant differences (P<0.001) between the RRF+ and RRF− groups with regard to renal length, width, thickness and volume of the left (length, 7.9±1.2 vs. 6.8±1.2 cm; volume, 60.0±26.7 vs. 40.2±18.1 ml, respectively) and right (length, 7.6±1.2 vs. 6.7±1.2 cm; volume, 50.2±26.5 vs. 33.9±15.3 ml, respectively) sides of the kidney. Multivariable stepwise forward logistic regression analyses showed that the mean renal length or volume and hemodialysis duration were independent predictors of the presence of RRF. Therefore, renal size assessment by ultrasonography may be useful for RRF evaluation in patients undergoing chronic hemodialysis.

## Introduction

In patients undergoing maintenance hemodialysis, monitoring the residual renal function (RRF) is clinically important (1). RRF not only reflects the remaining glomerular filtration rate (GFR), but also indicates the remaining endocrine functions, including erythropoietin production (2), calcium, phosphorus and vitamin D homeostasis (3,4), volume control and the removal of ‘middle molecules’ or low molecular weight proteins (5,6). RRF contributes to the adequacy of dialysis (7), quality of life (7) and reduced mortality rate (8) of end-stage renal disease (ESRD) patients.

However, the measurement and monitoring of RRF remains a challenge. With regard to ESRD, no ideal laboratory tests of renal function are available for assessing the manifestation of RRF compared with normal or early stage renal disease. The gold standard of renal function assessment is the measurement of GFR (9). Inulin clearance and radioisotopic methods used to measure GFR are expensive, complicated and not always available (10). The average creatinine clearance (C_Cr_) and urea clearance (C_urea_) have been recommended for ESRD assessment (11), however, these procedures require 24 h urine collection, which is not always possible in dialysis patients. The Modification of Diet in Renal Disease formula may be used when accurate urine collection is not possible, but the formula is likely to produce a marked overestimation of the GFR in dialysis patients (11). The majority of doctors select urine volume <200 ml/24 h as the indicator of RRF loss due to its simplicity and feasibility (11–17). However, this does have apparent shortcomings, including inaccuracy, difficulties in urine collection and its non-individualized nature.

Evaluation of renal size by sonography has become common practice when treating patients with kidney disease (18). A reduction in renal size has been used as an indicator of chronic kidney diseases (CKDs) (19). Furthermore, according to certain studies, it is possible to use renal size to predict the renal function in healthy individuals or patients with CKD (10,19,20). This may be useful when existing laboratory tests are not effective or suitable for certain circumstances, including the evaluation of older patients (20) and patients receiving a transplant (10). The most commonly used parameters of renal size are renal length and volume (19), although certain individuals prefer the renal size/body height (18) and renal volume/body surface area ratios (19).

With regard to the difficulties that arise when using traditional indices in RRF evaluation and the documented association between ultrasound renal size and renal function, we hypothesize that renal sonography is likely to provide useful information on RRF. However, to date, it is not known whether a correlation exists between renal size and the presence or absence of RRF in patients undergoing chronic hemodialysis. Therefore, the aim of the present study was to evaluate the correlation between sonographic renal size and RRF.

## Materials and methods

### Patient population

Patients were recruited from the Hemodialysis Center of the PLA 309th Hospital (Beijing, China). Patients with ESRD who were receiving regular hemodialysis three times a week and agreed to participate in the study were recruited. Patients undergoing hemodialysis for <3 months or with polycystic kidneys, a unilateral kidney, hydronephrosis or other abnormalities that significantly affected their renal size, including tuberculosis, neoplasm and malformation, were excluded from the study. The inclusion or exclusion of patients was determined by doctors from the Department of Nephrology by reviewing the medical records prior to further measurement. Clinical characteristics of the patients, including gender, age, height, weight, underlying diseases and duration of hemodialysis, were recorded. Body surface area was determined using Mosteller’s simplified equation. All measurements were recorded following a dialysis session to minimize any distortion caused by excess tissue fluid. Interdialysis urine collections (48 h) were used to calculate the daily urine volume (14). Patients were asked to begin urine collection by voiding following dialysis and to discard the first specimen (13). All urine was collected thereafter, with the final urine collection prior to the beginning of the next dialysis session (13). During this period, the patients were asked to eat and drink as usual. Patients were divided into two groups according to the average daily urine volume. RRF+ patients had a daily urine volume ≥200 ml/24 h (n=65; male, 31; female, 44), while RRF− patients had a daily urine volume <200 ml/24 h (n=75; male, 26; female, 39; Table I). The study was approved by the institutional review board of the PLA 309th Hospital and informed consent was provided by all participants.

### Renal size measurement

Renal sizes were measured by sonography on fasting subjects with empty bladders using a ProSound SSD Alpha 10 scanner (Hitachi, Aloka, Tokyo, Japan) equipped with a 2–6 MHz convex probe. Examinations were performed 24 h after a hemodialysis session in the interdialytic period by the same experienced examiner. Examinations started with the patients in a supine position. Next, the patients were scanned from a lateral, posterolateral or posterior view (in a prone position), according to which approach enabled optimal visualization of the kidney. Renal length was defined as the maximum longitudinal length. The accuracy of the electronic calipers in the ultrasound machine was 1 mm. The maximum length of the kidney was measured in the sagittal plane and the width and thickness of the kidney were measured in the transverse plane. All the measurements were performed in triplicate and the arithmetic mean was obtained. Renal volume was calculated with the ellipsoid formula: volume = length × width × thickness × π/6, where volume was measured in milliliters. The renal size/body height and renal volume/body surface area ratios were calculated. Mean renal length and volume were calculated as follows: (left + right)/2.

### Statistical analysis

Data processing and statistical analysis were performed using Excel software (Microsoft, Redmond, WA, USA) and SPSS software version 13.0 (SPSS, Inc., Chicago, IL, USA). Data are expressed as the mean ± SD. Categorical variables were compared using the χ^2^ test and continuous variables were compared with the Student’s t-test. To further characterize the correlation between renal length or volume and the presence/absence of RRF, univariate and multivariate stepwise forward logistic regression analysis was performed between the presence of RRF and all other variables. P<0.05 was considered to indicate a statistically significant difference.

## Results

### Clinical characteristics

Clinical characteristics of the two groups are presented in Table I. There were no statistically significant differences identified in gender, age and height of the patients between the two groups (P>0.05). In the RRF− group, weight and body surface area were significantly lower (P<0.05) and the duration of hemodialysis was significantly longer (P<0.001). The most common underlying disease was chronic glomerulonephritis, followed by diabetic nephropathy and aristolochic acid nephropathy for the two groups. No significant difference in the underlying diseases was identified between the two groups.

### Renal parameters

**Figure 1:**
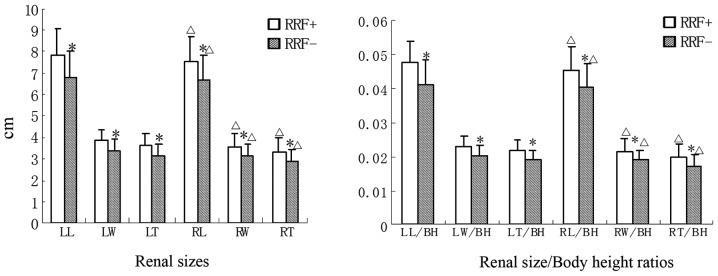
Sonographic renal sizes and the renal size/body height ratios of patients in the RRF+ and RRF− groups. ^*^P<0.001, vs. RRF+; ^△^P<0.001, vs. left kidney. LL, left length; LW, left width; LT, left thickness; RL, right length; RW, right width; RT, right thickness; BH, body height; RRF, residual renal function.

**Figure 2:**
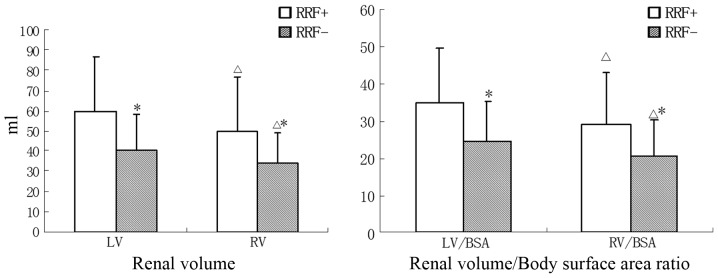
Renal volumes and the renal volume/body surface area ratios of the left and right kidneys in patients in the RRF+ and RRF− groups.^*^P<0.001, vs. RRF+; ^△^P<0.001, vs. left kidney. LV, left renal volume; RV, right renal volume; BSA, body surface area; RRF, residual renal function.

Sonographic renal sizes are shown in Figs. 1 and 2. There were statistically significant differences between the RRF+ and RRF− groups with regard to renal length of the left (7.9±1.2 vs. 6.8±1.2 cm, respectively) and right kidneys (7.6±1.2 vs. 6.7±1.2 cm, respectively), the width of the left (3.8±0.6 vs. 3.4±0.6 cm, respectively) and right kidneys (3.6±0.6 vs. 3.1±0.5 cm, respectively), the thickness of the left (3.6±0.6 vs. 3.1±0.5 cm, respectively) and right kidneys (3.3±0.6 vs. 2.9±0.5 cm, respectively) and the renal volume of the left (60.0±26.7 vs. 40.2 ± 18.1 ml, respectively) and right kidneys (50.2±26.5 vs. 33.9±15.3 ml, respectively) (all P<0.001). In the RRF− group, the left and right kidneys were on average 1.1 cm (13.6%) and 0.9 cm (11.3%) shorter in length and 19.8 ml (32.9%) and 16.4 ml (32.6%) smaller in volume, respectively, compared with those in the RRF+ group. There were also significant differences between the two groups in the renal size/body height and renal volume/body surface area ratios (P<0.001, for all). All the parameters for the left kidney were significantly higher compared with those for the right kidney (P<0.001, for all).

### Logistic regression analysis

**Figure 3:**
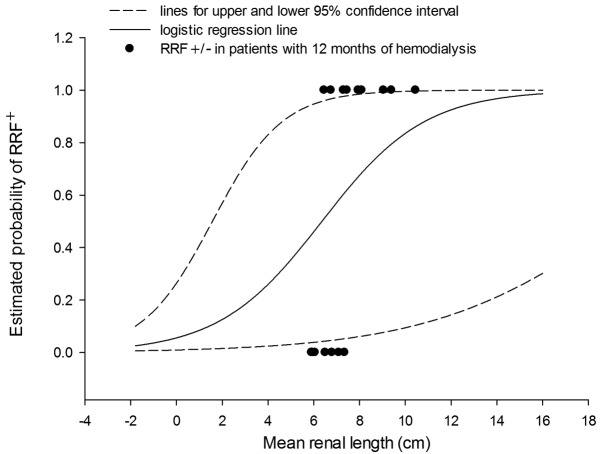
Estimated probability of RRF as a function of renal length, based on multiple logistic regression analysis in patients undergoing 12 months of hemodialysis. Solid/open circles indicate the presence/absence of RRF in patients undergoing 12 months of hemodialysis. RRF, residual renal function.

**Figure 4:**
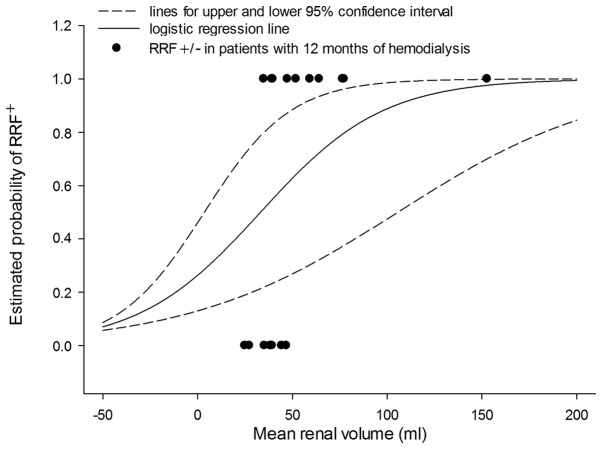
Estimated probability of RRF as a function of renal volume, based on multiple logistic regression analysis in patients undergoing 12 months of hemodialysis. Solid/open circles indicate the presence/absence of RRF in patients undergoing 12 months of hemodialysis. RRF, residual renal function.

**Figure 5:**
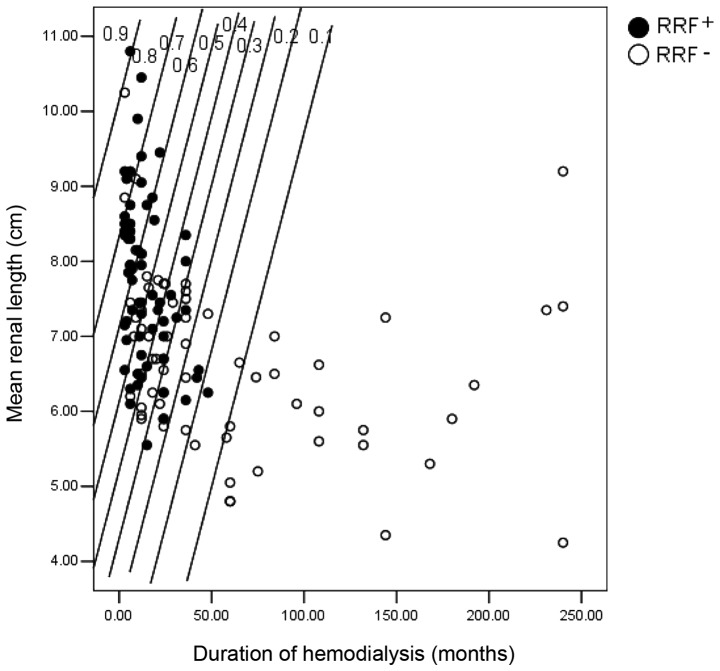
Scatter plot of mean renal length against the duration of hemodialysis. Solid circles represent the patients with RRF and open circles represent the patients without RRF. The straight lines are the contours of constant probability of RRF, estimated with the logistic regression model. RRF, residual renal function.

**Figure 6:**
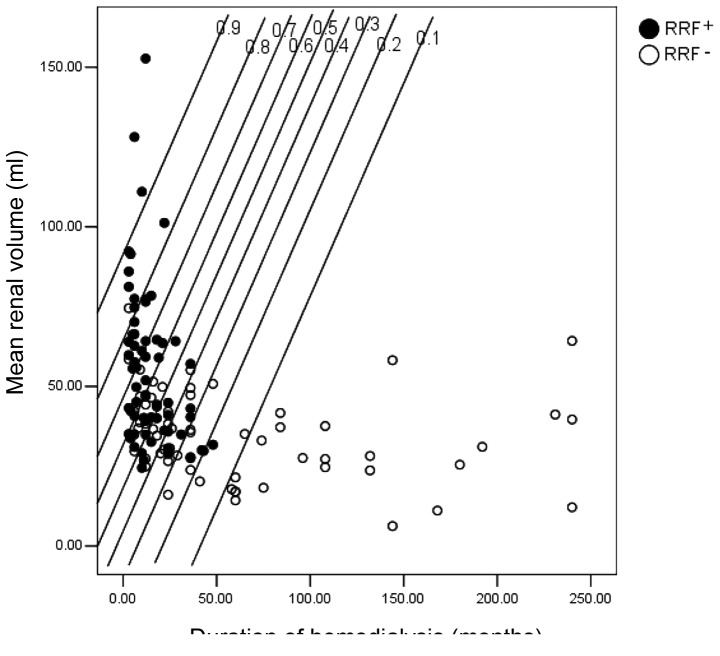
Scatter plot of mean renal volume against the duration of hemodialysis. Solid circles represent the patients with RRF and open circles represent the patients without RRF. The straight lines are the contours of constant probability of RRF, estimated with the logistic regression model. RRF, residual renal function.

Univariable and multivariable logistic regression analysis, using the presence of RRF as the dependent variable, was performed to identify independent associated factors (Table II). The mean renal length and volume were analyzed separately in the multivariate analysis, since renal volume was calculated from renal length. With the univariable analysis, weight, body surface area, mean renal length, mean renal volume and the duration of hemodialysis were associated with the presence of RRF. However, in multivariate stepwise forward analysis, only mean renal length or volume and the duration of hemodialysis were significantly associated with the presence of RRF. Figs. 3 and 4 were plotted based on the estimated probability of RRF against renal length and volume, respectively, using the aforementioned multiple logistic regression model in patients undergoing 12 months of hemodialysis. Scatter plots were also created based on the mean renal length and volume against the duration of hemodialysis, in Figs. 5 and 6, respectively. The contours show the constant probability of RRF estimated using the logistic regression model.

## Discussion

To the best of our knowledge, there have been no studies published on the correlation between renal size and the occurrence of RRF in patients undergoing chronic hemodialysis. For the first time, the results of the present study show that renal size is correlated with the development of RRF in patients undergoing chronic hemodialysis. In addition, it is possible to use renal length or volume as an independent predictor of the presence of RRF. These observations may aid clinicians in the evaluation of RRF in patients undergoing chronic hemodialysis.

The observations of the current study are consistent with previously reported results, which indicate that renal size correlates with renal function. The study by Sanusi *et al* (19) showed that renal length and volume correlate well with measured and predicted GFRs in a group of patients with CKD, which strengthened the usability of renal length in predicting the GFR. Paleologo *et al* (10) indicated that the renal dimensions measured by sonography correlate with GFRs in renal transplant recipients and kidney donors, while the accuracy of renal length as an indicator of GFR impairment was not statistically different from laboratory tests. Kim *et al* (21) found that there were statistically significant differences between the normalized total renal volumes in the various stages of renal function, and that the volumes decreased with renal function stage. The study by Adibi *et al* (22) showed that ultrasonographic renal size, particularly renal length, correlates with GFR in healthy children. Van Den Noortgate *et al* (20) identified that there was a significant correlation between renal mass and GFR in older patients. All these studies indicate that changes in renal size reflect a change in renal function. In the present study, these observations were confirmed in a special patient population and the results showed that renal size is associated with RRF in patients undergoing chronic hemodialysis.

The renal size of the patients in the current study was much lower compared with that of the patients in the study by Sanusi *et al* (19). This difference may stem from differences in the patient populations. The patients in the previous study also had decreased GFRs, however, the authors included patients in all stages of CKD. By contrast, the current study only included patients with ≥3 months of hemodialysis, which represents the final stage of CKD and the later span of renal shrinkage. Kim *et al* (21) examined renal volume according to the stage of CKD and obtained similar results to the current study with regard to renal volume of stage 5 CKD. However, in the earlier study, patients were not divided by RRF status.

Size difference between the left and right kidney has been increasingly studied (23,24). The results of the present study indicated that the left kidney was significantly larger than the right kidney in all parameters of renal size in the two groups. This observation is consistent with that of the majority of studies (23,24), which show that in healthy people, the left kidney is usually larger than the right kidney. This difference may be caused by the left kidney having a relatively larger space to grow, since the spleen is smaller than the liver. Additionally, the left renal artery is shorter and straighter than the right; thus, increased blood flow in the left artery may result in increased volume (23,24). The current results demonstrate that this trend was maintained at the final stage during kidney impairment.

Renal length and volume are recommended for renal size evaluation (19,25). In the present study, renal length and volume decreased in patients without RRF and were shown to independently predict the presence of RRF. Renal length is measured directly during ultrasound scanning and does not require geometrical assumptions or calculations that are required to assess renal volume. The ratios of renal size/body height and renal volume/body surface area were also evaluated in the present study, as it has been reported that renal length correlates with body height/renal volume and body surface area (19), and significant differences between the groups were observed. The ratios may be useful for evaluating patients with highly varied anthropometric data. However, renal length is the most convenient and direct variable for evaluating renal size in clinical practice.

However, the present study had several limitations. Firstly, the cross-sectional and observational nature of the analysis only described the associations observed. Secondly, it is not as accurate to use 200 ml daily urine volume as a threshold to define the status of RRF. Measurement of daily urine volume may be inaccurate without bladder catheterization when the urine volume is very low. In addition, the threshold of daily urine volume to define loss of RRF should be individualized to prevent over- or underestimations of RRF for specific patients. Although the majority of authors set the threshold as 200 ml arbitrarily (11–17), others use <100 ml (26) or <400 ml (27) to define the absence of RRF. Future studies should use the more complicated, but highly recommended method of determining the average C_Cr_ and C_urea_, which can be used to evaluate RRF quantitatively rather than qualitatively. Finally, ultrasound was used for the measurement of renal length and volume in the present study, while it is now increasingly recognized that the more expensive magnetic resonance imaging is superior to ultrasound in determining renal length and volume (25). Our sample size is modest since this is only a one center study. We are planning to include more centers and more patients for further study. In conclusion, sonographically determined kidney length and kidney volume may be used to predict the presence and absence of RRF in chronic hemodialysis patients. However, improved methods and a larger sample are required to further evaluate this topic.

## Figures and Tables

**Table I:** Clinical characteristics of the patients.

Clinical characteristics	Group RRF+	Group RRF−	P-value
Cases, n	65	75	
Age, years	51.4±15.5	52.0±16.6	0.874
Gender, male/female	31/44	26/39	0.873
Height, cm	166.3±7.7	166.5±7.3	0.858
Weight, kg	63.6±11.1	59.2±9.8	0.015
Body surface area, m^2^	1.71±0.21	1.65±0.16	0.041
Underlying diseases, n (%)			
Chronic glomerulonephritis (type unspecified)	36 (55.4)	47 (62.7)	0.382
IgA nephropathy	2 (3.1)	6 (8)	0.211
Diabetic nephropathy	13 (20)	10 (13.3)	0.288
Aristolochic acid nephropathy	7 (10.8)	6 (8)	0.573
Hypertension	4 (6.2)	2 (2.7)	0.310
Lupus nephritis	1 (1.5)	1 (1.3)	
Henoch-Schonlein purpura nephritis	1 (1.5)	1 (1.3)	
Obstructive nephropathy	0	1 (1.3)	
Analgesic nephropathy	1 (1.5)	1 (1.3)	
Duration of hemodialysis, months	14.5±11.3	56.1±62.4	<0.001

RRF, residual renal function. Quantitative data are presented as the mean ± standard deviation.

**Table II:** Univariate and multivariate stepwise forward logistic regression analysis between the presence of RRF and other variables, with renal length or volume as one of the independent variables.

A, Mean renal length, cm				

	Univariate		Multivariate (stepwise forward)	
		
Variables	β-value	P-value	β-value	P-value
Age	−0.003	0.804		
Male gender	0.055	0.873		
Height	−0.004	0.856		
Weight	0.041	0.017		
Body surface area	2.151	0.044		
Mean renal length	0.762	<0.001	0.445	0.033
Duration of hemodialysis	−0.054	<0.001	−0.042	0.002
Constant	−2.322	0.156		

B, Mean renal volume, ml				

	Univariate		Multivariate (stepwise forward)	
		
Variables	β-value	P-value	β-value	P-value

Age	−0.003	0.804		
Male gender	0.055	0.873		
Height	−0.004	0.856		
Weight	0.041	0.017		
Body surface area	2.151	0.044		
Mean renal volume	0.050	<0.001	0.031	0.013
Duration of hemodialysis	−0.054	<0.001	−0.040	0.002
Constant	−0.547	0.445		

RRF, residual renal function.

## References

[1] Brener ZZ, Kotanko P, Thijssen S (2010). Clinical benefit of preserving residual renal function in dialysis patients: an update for clinicians. Am J Med Sci.

[2] Artunc F, Risler T (2007). Serum erythropoietin concentrations and responses to anaemia in patients with or without chronic kidney disease. Nephrol Dial Transplant.

[3] Viaene L, Bammens B, Meijers BK (2012). Residual renal function is an independent determinant of serum FGF-23 levels in dialysis patients. Nephrol Dial Transplant.

[4] Wang AY, Lam CW, Wang M (2009). Is valvular calcification a part of the missing link between residual kidney function and cardiac hypertrophy in peritoneal ddialysis patients?. Clin J Am Soc Nephrol.

[5] Thomas J, Teitelbaum I (2011). Preservation of residual renal function in dialysis patients. Adv Perit Dial.

[6] Gerhardt T, Pöge U, Stoffel-Wagner B (2008). Serum levels of beta-trace protein and its association to diuresis in haemodialysis patients. Nephrol Dial Transplant.

[7] Perl J, Bargman JM (2009). The importance of residual kidney function for patients on dialysis: a critical review. Am J Kidney Dis.

[8] van der Wal WM, Noordzij M, Dekker FW, Netherlands Cooperative Study on the Adequacy of Dialysis Study Group (NECOSAD) (2011). Full loss of residual renal function causes higher mortality in dialysis patients; findings from a marginal structural model. Nephrol Dial Transplant.

[9] Frank M, Guarino-Gubler S, Burnier M (2012). Estimation of glomerular filtration rate in hospitalised patients: are we overestimating renal function?. Swiss Med Wkly.

[10] Paleologo G, Abdelkawy H, Barsotti M (2007). Kidney dimensions at sonography are correlated with glomerular filtration rate in renal transplant recipients and in kidney donors. Transplant Proc.

[11] Moist LM, Port FK, Orzol SM (2000). Predictors of loss of residual renal function among new dialysis patients. J Am Soc Nephrol.

[12] Suda T, Hiroshige K, Ohta T (2000). The contribution of residual renal function to overall nutritional status in chronic haemodialysis patients. Nephrol Dial Transplant.

[13] Erkan E, Moritz M, Kaskel F (2001). Impact of residual renal function in children on hemodialysis. Pediatr Nephrol.

[14] Richardson D, Lindley EJ, Will EJ (2003). A randomized, controlled study of the consequences of hemodialysis membrane composition on erythropoietic response. Am J Kidney Dis.

[15] Dervisoglu E, Altun EA, Kalender B, Caglayan C (2007). Effects of residual renal function on clinical and laboratory features of patients on continuous ambulatory peritoneal dialysis. BANTAO Journal.

[16] Bragg-Gresham JL, Fissell RB, Mason NA (2007). Diuretic use, residual renal function, and mortality among hemodialysis patients in the Dialysis Outcomes and Practice Pattern Study (DOPPS). Am J Kidney Dis.

[17] Khalil AA, Frazier SK, Lennie TA, Sawaya BP (2011). Depressive symptoms and dietary adherence in patients with end-stage renal disease. J Ren Care.

[18] Miletić D, Fuckar Z, Sustić A (1998). Sonographic measurement of absolute and relative renal length in adults. J Clin Ultrasound.

[19] Sanusi AA, Arogundade FA, Famurewa OC (2009). Relationship of ultrasonographically determined kidney volume with measured GFR, calculated creatinine clearance and other parameters in chronic kidney disease (CKD). Nephrol Dial Transplant.

[20] Van Den Noortgate N, Velghe A, Petrovic M (2003). The role of ultrasonography in the assessment of renal function in the elderly. J Nephrol.

[21] Kim HC, Yang DM, Jin W, Lee SH (2010). Relation between total renal volume and renal function: usefulness of 3D sonographic measurements with a matrix array transducer. AJR Am J Roentgenol.

[22] Adibi A, Adibi I, Khosravi P (2007). Do kidney sizes in ultrasonography correlate to glomerular filtration rate in healthy children?. Australas Radiol.

[23] Emamian SA, Nielsen MB, Pedersen JF, Ytte L (1993). Kidney dimensions at sonography: correlation with age, sex, and habitus in 665 adult volunteers. AJR Am J Roentgenol.

[24] Brandt TD, Neiman HL, Dragowski MJ (1982). Ultrasound assessment of normal renal dimensions. J Ultrasound Med.

[25] Cheong B, Muthupillai R, Rubin MF, Flamm SD (2007). Normal values for renal length and volume as measured by magnetic resonance imaging. Clin J Am Soc Nephrol.

[26] Shemin D, Bostom AG, Laliberty P, Dworkin LD (2001). Residual renal function and mortality risk in hemodialysis patients. Am J Kidney Dis.

[27] Hetakeyama S, Abe A, Suzuki T (2011). Clearance and safety of the radiocontrast medium iopamidol in peritoneal dialysis patients. Int J Nephrol.

